# Communicating and Dealing with Uncertainty in General Practice: The Association with Neuroticism

**DOI:** 10.1371/journal.pone.0102780

**Published:** 2014-07-16

**Authors:** Antonius Schneider, Magdalena Wübken, Klaus Linde, Markus Bühner

**Affiliations:** 1 Institute of General Practice, Klinikum rechts der Isar, Technische Universität München, München, Germany; 2 Department of Psychology, Ludwig-Maximilians-Universität München, München, Germany; Nagoya University Graduate School of Medicine, Japan

## Abstract

**Background:**

Diagnostic reasoning in primary care setting where presented problems and patients are mostly unselected appears as a complex process. The aim was to develop a questionnaire to describe how general practitioners (GPs) deal with uncertainty to gain more insight into the decisional process. The association of personality traits with medical decision making was investigated additionally.

**Methods:**

Raw items were identified by literature research and focus group. Items were improved by interviewing ten GPs with thinking-aloud-method. A personal case vignette related to a complex and uncertainty situation was introduced. The final questionnaire was administered to 228 GPs in Germany. Factorial validity was calculated with explorative and confirmatory factor analysis. The results of the Communicating and Dealing with Uncertainty (CoDU) – questionnaire were compared with the scales of the ‘Physician Reaction to Uncertainty’ (PRU) questionnaire and with the personality traits which were determined with the Big Five Inventory (BFI-K).

**Results:**

The items could be assigned to four scales with varying internal consistency, namely ‘communicating uncertainty’ (Cronbach alpha 0.79), ‘diagnostic action’ (0.60), ‘intuition’ (0.39) and ‘extended social anamnesis’ (0.69). Neuroticism was positively associated with all PRU scales ‘anxiety due to uncertainty’ (Pearson correlation 0.487), ‘concerns about bad outcomes’ (0.488), ‘reluctance to disclose uncertainty to patients’ (0.287), ‘reluctance to disclose mistakes to physicians’ (0.212) and negatively associated with the CoDU scale ‘communicating uncertainty’ (−0.242) (p<0.01 for all). ‘Extraversion’ (0.146; p<0.05), ‘agreeableness’ (0.145, p<0.05), ‘conscientiousness’ (0.168, p<0.05) and ‘openness to experience’ (0.186, p<0.01) were significantly positively associated with ‘communicating uncertainty’. ‘Extraversion’ (0.162), ‘consciousness’ (0.158) and ‘openness to experience’ (0.155) were associated with ‘extended social anamnesis’ (p<0.05).

**Conclusion:**

The questionnaire allowed describing the diagnostic decision making process of general practitioners in complex situations. Personality traits are associated with diagnostic reasoning and communication with patients, which might be important for medical education and quality improvement purposes.

## Introduction

Dealing with uncertainty is a core element in the provision of care in general practice [1]. This can be explained by systemic and epidemiological considerations. The systemic argument was developed by Ravetz, who asserts that increasing knowledge and fortress generates increasing ignorance, which in turn makes scientific assessments more complex [2]. Wheling shows that these boundaries of knowledge are also challenging for individual medical treatment decisions [3]. Hence the field of medical knowledge enlarges the boundaries of ignorance and the accompanying uncertainty at the same time. Epidemiological studies investigating the selection process of patients across different sectors of health care illustrate the lower pre-test probability of individual diseases in primary care when compared with the hospital setting [4]. This lower pre-test probability implies low positive predictive values when interpreting the results of diagnostic procedures (i.e. the probability that someone with a positive test result is really ill) [5]. Furthermore, patients are coming with the first symptoms and thus often with lower severity of their disease to their general practitioner (GP) [6], and diagnostic reasoning needs to take into account the holistic bio-psycho-social context to meet the needs of the patients adequately. The resulting uncertainty, which is related to the large variety of possible diagnoses, represents a challenge to general practitioners in particular [1].

The question that arises is about how GPs deal with this inevitable uncertainty in their daily practice and if a better understanding of the above-mentioned relationships might help to improve the quality of care. Several attempts have been made to unravel the emotional and cognitive aspects of this issue. Gerrity et al. were the first to develop a validated questionnaire that measures the affective reaction to uncertainty [7], [8]. This questionnaire demonstrated that higher anxiety due to uncertainty is associated with higher resource use [9]. Stolper et al. developed a questionnaire that measures the impact of gut feelings on medical decision-making in terms of ‘sense of alarm’ and ‘sense of reassurance’ [10]. These aspects are coming close to heuristics, which seem to play an important role for GPs. Heuristic medical decisions are often made unconsciously and thus attributed to intuition. Intuition might be understood as a cognitive ‘short-circuiting’ where a decision is reached even though the reasons for the decision cannot easily be described [11]. Various investigations highlight the impact of heuristics for medical decision-making [12], [13]. However, there is a strong debate about the usefulness of ‘heuristics’ and intuition [14] as these may sometimes lead to exceptional results but also to fatal errors [15], [16]. Beyond this, the nature of intuition in general practice is still unclear.

Another important aspect is that personality characteristics might influence diagnostic decision-making, as previous studies have shown some relationships between cognition and personality traits. For example, neuroticism was linked to lower performance across various domains including information processing, pattern analysis and memory; and extraversion was shown to be related with creativity, speed, long-term memory, but was negatively associated with reasoning [17]–[19]. Therefore, personality characteristics like neuroticism or extraversion might influence the way how GPs deal with uncertainty.

In 2009, the first author introduced the ‘Dealing with Uncertainty Questionnaire’ (DUQ), which allowed for the describing of some of these cognitive aspects in more detail [20]. For instance, it could be shown that the item ‘test of time’ was associated with the item ‘intuition plays a certain role,’ which might contribute to a simple heuristic in keeping with the Bayes’ Theorem. However, the internal consistency of this ‘GP heuristic scale’ was low. The aim of this study was to improve the psychometric properties of the DUQ or to revise the questionnaire, if necessary; and to estimate the association with personality traits on handling of uncertain situations in general practice.

## Methods

### Study design

The study used both qualitative and quantitative methods. In a first step, the original version of the DUQ was discussed within an international focus group of 6 experienced GPs and psychologists (4 women, 2 men) from Portugal, Italy, Austria, Denmark and Germany, who participated in a workshop named ‘Dealing with diagnostic uncertainty’ at the EQuiP Invitational Conference in 2011. The focus group was videotaped. A qualitative analysis of content was performed by MW and AS. The participants of the focus group discussion gave verbal informed consent for video recording and analysis. In a second step, new raw items were generated based on the results of the focus group and literature research. In a third step, the draft version of the questionnaire was presented to experienced GPs. They had to fill in the questionnaire by thinking out loud, which we used to uncover inconsistencies, missing items, or items which might be difficult to understand. The interviews were audiotaped. Inconsistencies and needs for item improvement derived from cognitive think aloud technique were identified by MW, MB and AS. The cognitive think aloud technique is seen as an optimal method to capture thought processes [21]. The questionnaire was improved in an iterative development process, following the suggestions of the general practitioners.

Finally, we performed a cross-sectional survey with GPs. Participants were recruited at conferences in Germany or from the network of teaching practices of the University Hospital Klinikum rechts der Isar. They were asked to fill in an anonymised questionnaire. The study was approved by the Medical Ethics Committee of the Technische Universität München/University Hospital Klinikum rechts der Isar. The participants of the individual interviews and of the survey gave written informed consent. The study was carried out between April 2011 and March 2013.

### Additional questionnaires

#### Physicians’ affective Reaction to Uncertainty (PRU)

Gerrity et al. developed a questionnaire to measure physicians’ affective reaction to uncertainty [7], the PRU, which was revised in 1995 [8]. The German version of the PRU was validated and culturally adapted in 2007 with satisfying psychometric properties [22]. The German version of the PRU consists of four scales named ‘anxiety due to uncertainty’ (items 1–5), ‘concern about bad outcomes’ (items 6–8), ‘reluctance to disclose uncertainty to patients’ (items 9–13) and ‘reluctance to disclose mistakes to physicians’ (items 14–15). The items are rated on a 6-point Likert scale: 1 = strongly disagree, 2 = moderately disagree, 3 = slightly disagree, 4 = slightly agree, 5 = moderately agree, 6 = strongly agree. The scales are scored by summing physicians’ responses to each item in the scale (items 4, 9, 10 and 12 are reverse scored) with a maximum toleration of one item missing in the scales. The greater the score, the greater the physicians’ affective reaction to uncertainty at that scale.

#### Assessment of personality traits: Big Five Inventory (BFI-K), short version

The structure of personality traits, particularly the five-factor model of personality, emerges quite consistently across cultures [23] and is widely accepted for research on job performance and in the area of medicine [24], [25]. The five-factor model comprises the following personality traits [25]:

Neuroticism: The negative pole of emotional stability describes an individual’s tendency to become emotionally upset. Common attributes are anxiety, depression, anger, self-consciousness, low self-esteem, embarrassment, worry, fearfulness, instability and insecurity. Neuroticism is associated with vulnerable and employ maladaptive coping strategies in dealing with stressful situations.
Extraversion: A person’s capacity for joy and the tendency to seek interpersonal stimulation. Common attributes are traits relating to sociability and dominance and to being energetic, active, talkative, fun-loving, gregarious and persuasive. Seek situations where they can interact with others.
Openness: Conceptualised as influencing the breadth and complexity of mental experiences of individuals. Common attributes are being imaginative, curious, original, broadminded, and intellectual, creative, insightful and perceived as more intelligent by others.
Agreeableness: The tendency to help others and behave in pro-social ways. Common attributes are: cooperative, nurturing, sensitive, caring, altruistic, kind, tender-minded and soft-hearted. Individuals scoring low are described as uncooperative, unfriendly, selfish, hostile and egocentric.
Conscientiousness: Associated with achievement-striving, prudence, dependability, persistence, order, careful planning, gratification delay and impulse control. Associated with the tendency to work hard and goal-directed behaviour. Tend to follow rules and plan carefully, ability to delay gratification.

The BFI-K was developed for an efficient way to assess the five factors (“Big Five”) of personality with satisfying psychometric properties. The advantage of the short version is that it takes only about two minutes to fill in when maintaining good test properties [26]. It consists of 21 items evenly distributed to the scales ‘extraversion’ (4 items), ‘agreeableness’ (4 items), ‘conscientiousness’ (4 items), ‘neuroticism’ (4 items) and ‘openness to experience’ (5 items). The items are rated on a 5-point Likert scale: 1 = fully inapplicable, 2 = rather inapplicable, 3 = neither nor, 4 = rather applicable, 5 = fully applicable. Higher scoring correlates with a stronger accentuation of the respective personality trait. The average scale scores (some items are reverse scored) were generated with a maximum toleration of one item missing per scale. The questionnaire is especially useful for the comparison of different groups and correlation analysis in research.

Additionally, demographic data was documented with respect to age, gender, years of clinical experience, years in private practice and type of work in private practice.

### Analysis

Data was analysed using the SPSS statistical package (version 21) and Mplus. Descriptive analysis included mean and standard deviation; differences were calculated with variance analysis (ANOVA). The associations between the scales of PRU, CoDU and the BFI-K were assessed with Pearson correlations (bootstrap, two-tailed). To explore the construct validity of the CoDU, we conducted an explorative factor analysis with the weighted least squares means and variances adjusted (WLSMV) estimation method available in MPlus, which we used because the Likert-type scales led to ordinal data. We applied a promax rotation, because the occurring factors were assumed to be correlated. The criterion for factor extraction was based on the results of the parallel analysis, which compares the eigenvalues of the sample correlation matrix with those obtained from a random data correlation matrices of the same number of variables and sample size at the 95^th^ percentile. Afterwards a confirmatory factor analysis was assessed to confirm the model fit. To measure internal consistency we calculated Cronbach alpha for each scale.

## Results

### Development of the Communicating and Dealing with Uncertainty (CoDU) Questionnaire

First, it was discussed in the focus group if the development of a further questionnaire would make sense; and which aspects need to be taken into account for improvement of the precursor version (DUQ). It was suggested to include the patient as a partner in the decision-making process, and the importance of psychological, familial and social aspects of the patient were emphasized. The participants deemed the questionnaire as too globally focused and suggested a case vignette at the beginning. Different, vague situations would create different possibilities to deal with uncertainty. The participants demanded that individual case vignettes would help the GP to adapt the answers to the clinical situation which crossed his or her mind. It was suggested to introduce the phrase “Please think of a typical patient consultation in your daily medical work where diagnostic uncertainty was involved”. Secondly, the items were modified and new items were added by in-depth analysis of literature [1], [7], [8], [12], [20], [27]. Notably, the work of Hewson et al. [27] was helpful as they identified explicit strategies to deal with uncertain and complex problems in primary care.

Thirdly, the cognitive thinking aloud procedure revealed that some items were questions were formulated to vague; and that the introduction of case vignettes was too imprecise. Various recommendations were made to change the wording to improve the comprehensibility. Notably, it was criticised that physicians might not always differentiate between intuition and gut feeling. It was recommended to use of the phrase “first impression of the patient” instead. During this iterative development process, we had the impression that saturation was reached after interviewing 10 GPs, when no additional comments came up.

The definite version of the CoDU comprised 19 items after various adjustments during the development process (Table 1). This questionnaire was handed out in a last step on four GP conferences and to the network of teaching practices. Before filling in the items GPs were asked to report a case vignette and instructed to refer in their answers to this specific case. We asked the physicians if they would suggest additional possibilities to deal with uncertainty which have not been mentioned in the questionnaire.

**Table 1:** WLSMV factor analysis with promax rotated loadings of the CoDU (parallel analysis).

		Scale			
		1	2	3	4
4	I explained the exclusion of differential diagnoses to the patient in lay language.	**0.832**	−0.031	−0.145	−0.061
5	I discussed the therapeutic options with the patient.	**0.734**	−0.119	−0.156	−0.119
9	I assured that the patient understands the treatment plan which I have developed.	**0.700**	0.007	−0.001	−0.054
2	I explained the reasons for the symptoms of the possible spectrum of diseases in detail.	**0.681**	0.006	−0.054	0.211
6	I took a lot of time for the patient’s questions about the reason for encounter.	**0.560**	0.137	−0.065	−*0.315*
10	I assured that the patient’s personal circumstances enable the adherence of the treatment plan.	**0.554**	−0.037	0.191	−0.094
11	I balanced my working hypothesis against possible differential diagnosis.	**0.516**	0.042	0.090	−0.039
1	I told the patient clearly that there is still an uncertainty with respect to my working diagnosis.	**0.483**	−0.049	0.123	0.285
14	I discussed the available options with respect to the various courses of the disease with the patient.(e.g. “If the discomfort increases over the weekend, please go to the hospital”).	**0.444**	0.001	0.165	0.085
3	I took the fears of the patient into account when I excluded differential diagnoses.	**0.398**	0.143	0.115	−0.115
15	I referred the patient to a specialist for further diagnostic investigation.	−0.097	**0.866**	−0.159	−0.069
13	I arranged further investigations to prevent overlooking other potentially critical diagnoses.	0.116	**0.681**	0.017	0.181
19	The reason for encounter of my case vignette appeared as an urgent problem for me.	0.034	**0.457**	0.202	−0.040
12	I waited until the symptoms got clearer to alleviate my diagnostic decision(“watchful waiting, test of time”).	−0.029	−**0.445**	0.161	−0.046
17	The “first impression” of the patient played a major role for me in dealing with diagnostic uncertainty.	−0.123	−0.213	**0.766**	−0.055
16	My intuition played a major role in dealing with diagnostic uncertainty.	0.049	−0.007	**0.691**	0.042
18	It was of importance for me that the patient was “somehow different than usual”.	−0.247	0.270	**0.340**	−0.153
7	I included the family environment of the patient into my diagnostic considerations.	0.094	0.015	0.053	−**0.830**
8	I included the occupational environment of the patient into my diagnostic considerations.	0.066	−0.099	−0.008	−**0.664**
**Intern consistency - Cronbachs alpha**		.79	.60	.39	.69

1 = Communication with the patient, 2 = Diagnostic Action, 3 = Intuition, 4 = Psycho-social extension.

highest loadings are printed bold.

### Results of the cross-sectional survey

#### Sample characteristics

The demographic characteristic of participating GPs is summarized in Table 2. 228 physicians met the criteria and were included in the analysis. Seven questionnaires were excluded due to missing data. The participating men were significantly older (F = 9.58, eta^2^ = 0.04, p = 0.002), had more years of clinical experience (F = 9.28, eta^2^ = 0.04, p = 0.003) and more years in practice (F = 21.04, eta^2^ = 0.09, p<0.001). The GPs described the following clinical examples in their case vignettes: abdominal pain (n = 58 (25%)), chest pain (n = 32 (14%)), musculo-sceletal pain (n = 15 (7%)), unclear infection (n = 9 (4%)), fever of unclear origin (n = 8 (4%)), vertigo (n = 7 (3%)) and palpitation (n = 7 (3%)). The most vignettes described acute life-threatening conditions (like heart attack or appendicitis; n = 132; (58%)). Life-threating conditions which were delayed in time were the second most prevalent (like overlooked cancer; n = 65 (29%)). Conditions that were not life-threating (like strong sweating or pain in a finger; n = 26 (11%)) were lowest. It was not possible to estimate the possible hazard in 5 (2%) cases.

**Table 2:** Demographic Characteristics of the participating GPs.

	Total sample (N = 228)	Men (N = 146)	Women (N = 81)
Age M (SD)	51.5 (9.0)	52.9 (8.5)	49.1 (9.3)
Years of clinical experience M (SD)	23.9 (9.4)	25.2 (8.7)	21.3 (10.1)
Years in private practice M (SD)	16.6 (9.3)	18.6 (8.7)	12.7 (9.2)
Type of work in general medicine (%)			
Practitioner	93.0	96.6	86.4
Employed in a general practice	6.1	2.7	12.3
Retired	.9	.7	1.2

#### CoDU: Assessing scale internal consistency and factorial validity

The parallel analysis extracted 4 factors: Root 4: Eigenvalue 1.54> Random Data Eigenvalues at 95% percentile 1.35 and Root 5: Eigenvalue 1.17< Random Data Eigenvalues at 95% percentile 1.28. The random eigenvalue data course created exceeds the eigenvalue curse based on the number of items of the CoDU and participants of the study at root 5. Therefore 4 factors have to be extracted.

Table 3 shows the inter-correlations of the four CoDU scales with up to 0.3, meaning that the factors ‘communicating uncertainty’ and ‘intuition’ share a maximum of 9% of variance. We calculated a promax rotated explorative factor analysis (pattern matrix is shown in Table 1).

**Table 3:** Intercorrelations of the four CoDU - scales.

	Communicating uncertainty	Diagnostic action	Intuition	Extended social anamnesis
Communicating uncertainty	1.000			
Diagnostic action	0.276	1,000		
Intuition	0.300	0.264	1,000	
Extended social anamnesis	−0.112	−0.005	−0.163	1,000

Some items loaded on more than one defined scale. In these cases the highest loading led to the scale classification. The four extracted factors were ‘communicating uncertainty’ (10 items), ‘diagnostic action’ (4 items), ‘intuition’ (3 items) and ‘extended social anamnesis’ (2 items). The internal consistency of the scales measured with Cronbach alpha ranged from 0.39 (low) to 0.79 (acceptable). The structure of the questionnaire remained the same if only life-threatening cases (acute and chronic) were included in the factor analysis. The model fit was confirmed with a confirmatory factor analysis. A RMSEA of 0.074 in the exploratory factor analysis and 0.08 in the confirmatory factor analysis indicated an adequate model fit [28]. Thus, the RMSEA of the confirmatory factor analysis compared to the REMSEA of the exploratory factor analysis did only slightly change omitting cross loadings and specifying no error correlations. The scales were scored by summing doctors’ responses to each item of the scale (item 12 is reverse scored). Table 4 shows the descriptive statistics for the individual scales. The answers covered the full range, thus indicating the usefulness of the scaling. There were no notable floor or ceiling effects.

**Table 4:** Descriptive values of the CoDU questionnaire.

	Range	Min	Max	Mean	Median	SD	Floor	Ceiling
Communicating uncertainty (n = 227)	28.0	32.0	60.0	49.2	50.0	6.1	0%	3.5%
Diagnostic action (N = 228)	18.0	6.0	24.0	16.7	17.0	4.3	0%	6.6%
Intuition (n = 228)	15.0	3.0	18.0	11.4	11.0	3.4	1.3%	6.6%
Extended social anamnesis (n = 224)	10.0	2.0	12.0	8.0	8.0	2.9	5.7%	11.4%

The qualitative questions revealed two further topics which were not covered in the present questionnaire. The physicians suggested keeping contact by telephone as an additional strategy to deal with uncertainty in eight cases. Another strategy in thirteen situations was to present the patient to a colleague. There were no other suggestions going beyond the already named items.

#### Relations between personality traits and the uncertainty scales

Personality traits: ‘Anxiety due to uncertainty’ of the PRU scale correlated negatively with extraversion, conscientiousness and openness to experience, while being positively correlated with neuroticism (Table 5). The ‘communication uncertainty’ scale of the CoDU correlated with all personality traits. Notably, neuroticism correlated with all RRU scales and negatively with ‘communicating uncertainty,’ showing the strongest association in comparison with the other scales. ‘Extended social anamnesis’ correlated positively with extraversion, conscientiousness and openness to experience. However, the effect sizes were modest. The ‘diagnostic action’ and ‘intuition’ scales showed no significant associations.

**Table 5:** Pearson correlations between personality traits and uncertainty scales.

	Extroversion	Agreeableness	Conscien-tiousness	Neuroticism	Openness to experience
PRU-1: Anxiety due to Uncertainty	−0.198**	−0.040	−0.153*	0.487**	−0.134*
PRU-2: Concern about bad Outcomes	−0.125	0.071	−0.200**	0.488**	−0.014
PRU-3: Reluctance to Disclose Uncertainty to Patients	−0.054	−0.109	−0.020	0.287**	−0.155*
PRU-4: Reluctance to Disclose Mistakes to Physicians	−0.138*	−0.085	−0.120	0.212**	0.034
CoDU-1: Communi-cating uncertainty	0.146*	0.145*	0.168*	−0.242**	0.186**
CoDU-2: Diagnostic action	0.029	0.112	−0.009	−0.067	−0.020
CoDU-3: Intuition	0.000	0.057	−0.025	−0.053	−0.041
CoDU-4: Extended social anamnesis	0.162*	0.076	0.158*	−0.042	0.155*

Two-tailed test, **p<.01, *p<.05, results based on 1000 bootstrap – samples.

PRU = Physicians’ reactions to uncertainty questionnaire; CoDU = Communicating and dealing with uncertainty questionnaire.

## Discussion

Four scales emerged within the refined ‘Dealing with Uncertainty Questionnare’ from the development process using case vignettes. The items could be assigned to the scales ‘communicating uncertainty’, ‘diagnostic action’, ‘intuition’ and ‘extended social anamnesis’. Physicians scoring high in neurotisicm showed more anxiety due to uncertainty and higher reluctance to communicate with patients. Extraversion, conscientiousness and openness correlated negatively with anxiety due to uncertainty and positively with patient communication.

In comparison to the previous questionnaire [20], the communicative aspect evolved as a completely new scale. This effect might be explained by the introduction of the case vignettes, which allows the GPs to concentrate on their individual case, whereas the previous version of the DUQ was related to the general aspects about dealing with uncertainty. The importance of communication was already highlighted in several overviews or narrative reviews [1], [29]; and parts of the physicians’ attitudes regarding communciation are also named in the ‘reluctance to disclose uncertainty’ scale of the PRU [8]. The ‘diagnostic action’ scale was already developed in the previous DUQ version, but ‘extended social anamnesis’ and ‘intuition’ were summarised in one scale beforehand [20]. The introduction of more items, in particular those with respect to ‘first impression of the patient’ and ‘patient was somehow different than usual’ might have changed the assignments of the various items to the scales. As a consequence, two different scales were developed instead of extending the diagnostic reasoning towards including the psycho-social context of the patients and the more intuitional aspects of diagnostic reasoning.

The content validity appears to be high despite the mediocre internal consistency of the ‘diagnostic action’ and ‘intuition’ scales. Thus it was not possible to improve the psychometric properties compared to the previous version [20]. It might be speculated that the addition of more ‘action’ items might increase the internal consistency as it was 0.75 in the previous version. Unfortunately, in particular the ‘intution’ scale showed very low internal consistency. However, there is a debate about the usefulness of extending the questionnare solely to increase Cronbach alpha when the content validity is high [30]. We are sure that we have already achieved this by the repetitive involvement of many GPs during the development process. Generally, there is some difficulty to grasp the meaning of intuition. First, individual aspects of intuition might vary from person to person. Secondly, intuition might be valued differently by each GP. Heuristic decisions are often made intuitively, and fruitful heuristics have been worked out and their positive effects were demonstrated [13]. But heuristics and/or intution can also lead to fatal errors when important contextual information is ignored or alertness suppressed [14]. This might be explained by the dual cognitive process theory, where ‘System 2’ (i.e. reasoning that is slow, effortful and more conscious) might sometimes be too lax and allow many intuitive judgments (i.e. ‘System 1’, which is fast, effortless, associative, implicit and unconscious) to be expressed, including some that are erroneous [31]. The environment of the primary care setting with its patients presenting their whole bio-psycho-social breadth of problems seems very difficult to predict. Therefore it might hardly be possible to develop a consistent and generalisable intuition construct.

The validity of the ‘CoDU’ scales is underlined by the relations to different aspects of personality traits. The deep impact of the big five personality traits was demonstrated in the academic performance of medical students [25] and medical speciality choice [24]. It was revealed that extraversion and openness gained increasing importance in later academic performance, whereas conscientiousness was the most significant asset for all medical students [25]. GPs were characterised as sympathetic, trusting, cooperative and altruistic in the literature review of Borges et al. [24]. The authors found no cues with respect to neuroticism, neither for GPs nor for specialists. To our knowledge, this is the first study which evaluates the impact of personality traits on medical decision-making. In particular, neuroticism correlated negatively with a pro-active way to deal with uncertainty, including low willingness to communicate with the patients. Extraversion, conscientiousness and openess to experience were related to having a more constructive way to deal with uncertain situations, in particular with respect to communication. These relations might be important for medical education and vocational training. Establishing a good doctor-patient relationship, optimal communication skills in terms of shared medical decision-making [32] and a holistic bio-psycho-social approach are core values of modern healthcare professionals [33]. This might be in particular of importance when patients present with medically unexplained symptoms. These patients are often demanding repeatedly diagnostic investigations and various therapies without sustainable improvement of health status [34], [35]. Often pressure occurs in these situations going along with uncertainty on both sides, patients and doctors. It was worked out previously that optimal communication is necessary to avoid errors in communication, unnecessary investigation and somatic fixation [36], [37]. It might be speculated that accentuated personality traits of the doctor might hinder good communication during complex doctor-patient encounter. However, these findings might be incidental and thus need to be validated in further studies. Notwithstanding the homogenous pattern of correlations suggests a plausible association.

A further limitation of our findings might be explained by the fact that we developed and validated our questionnaire with motivated GPs who participated in medical conferences and often train medical students. These GPs might be more reflective and prone to critical thinking than the average GP. It remains unclear to what extent the internal consistency of the questionnaire might have been influenced by this fact. Certainly, the self-rating has some limitations in itself, as the questions could be answered due to social desirability. In order to test previous work [20] we have tried to saturate the different aspects of how GPs deal with uncertainty with an additional focus group and extensive evaluation. However, it was not possible to accomplish satisfying internal consistencies, which might be unachievable as stated above. Nevertheless, we believe that such questionnaires would benefit projects to understand medical decision-making in complex situations more in detail. In spite of some low reliability estimates the content validity is assured by elaborated construction process. Futhermore, it is not intended to use the scales for a psychometric single case diagnosis. The questionnaire should provide a rough overview about different strategies to deal with uncertainty and their relationship with individual characteristics. The strategies ‘keeping contact by telephone’ and ‘presenting the patient to a colleague’ were not identified during the focus group and ‘thinking aloud’ process. The first one might be assigned to the ‘communicating uncertainty’ scale, the latter to the ‘diagnostic action’ scale. Therefore it seems unlikely that the results related to the correlations between the CoDu and BFI-K scales are distorted. Applying confirmatory and exploratory factor analyses using the same sample is a limitation. Thus, the found structure needs to be cross validated with a different sample. A challenge was the variability of the presented case vignettes with regard to the kind of disease and severity, which was strongly suggested by the participants of the focus group. On the one hand, it stimulated the reporting of difficult situations on an individual level. On the other hand this could impede the comparability. It might be an implication for future research to evaluate strategies of dealing with uncertainty and their relations with personality traits using fixed case vignettes, in particular related to patients with medically unexplained symptoms.

## Conclusion

The CoDU-questionnaire emphasises the four constructs: ‘communicating with patients’, ‘diagnostic action’, ‘intuition’ and ‘extended social anamnesis’. Despite partially low internal consistency, the questionnaire was able to help analyse the diagnostic decision-making process of general practioners in complex situations with high content validity. Further research would be necessary to evaluate which kind of heuristics are useful in primary care settings and to determine how phyisicans’ personality characteristics might influence medical decision-making. Our questionnaire might help contribute to a better understanding of the way physicians deal with uncertainty. This would be useful for medical education and quality improvement purposes.
